# Screening of family members of chronic kidney disease patients with Fabry disease mutations: a very important and underrated task

**DOI:** 10.1590/2175-8239-JBN-2020-0080

**Published:** 2020-09-14

**Authors:** Luciana Senra de Souza Sodré, Rosália Maria Nunes Henriques Huaira, Fernando Antônio Basile Colugnati, Moises Carminatti, Luciane Senra de Souza Braga, Marcelo Paula Coutinho, Natália Maria da Silva Fernandes

**Affiliations:** 1 Universidade Federal de Juiz de Fora, Juiz de Fora, MG, Brasil.; 2 Faculdade de Medicina Campos, Campos dos Goytacazes, RJ, Brasil.

**Keywords:** Renal Insufficiency, Chronic, Fabry Disease, Triage, Family, Brazil, Insuficiência Renal Crônica, Doença de Fabry, Triagem, Família, Brasil

## Abstract

**Introduction:**

Fabry disease is a chronic, progressive, and multi-system hereditary condition, related to an Xq22 mutation in X chromosome, which results in deficiency of alpha-galactosidase enzyme, hence reduced capacity of globotriaosylceramide degradation.

**Objectives:**

to evaluate the prevalence of Fabry disease (FD) mutations, as well as its signs and symptoms, among relatives of chronic kidney disease (CKD) patients diagnosed with FD during a previously conducted study, named “Clinical and epidemiological analysis of Fabry disease in dialysis centers in Brazil”.

**Methods:**

a cross-sectional study was carried out, and data was collected by interviewing the relatives of patients enrolled in the Brazil Fabry Kidney Project and blood tests for both Gb3 dosage and genetic testing.

**Results:**

Among 1214 interviewed relatives, 115 (9.47%) were diagnosed with FD, with a predominance of women (66.10%). The most prevalent comorbidities were rheumatologic conditions and systemic hypertension (1.7% each), followed by heart, neurological, cerebrovascular diseases, and depression in 0.9% of individuals. Intolerance to physical exercise and tiredness were the most observed symptoms (1.7%), followed by periodic fever, intolerance to heat or cold, diffuse pain, burn sensation or numbness in hands and feet, reduced or absent sweating, as well as abdominal pain after meals in 0.9%.

**Conclusion:**

We found a prevalence of Fabry disease in 9.47% of relatives of CKD patients with this condition, remarkably with a 66.1% predominance of women, which contrasts with previous reports. The screening of family members of FD patients is important, since it can lead to early diagnosis and treatment, thus allowing better quality of life and improved clinical outcomes for these individuals.

## RESUMO

**Introdução:**

A doença de Fabry é uma condição hereditária crônica, progressiva e multissistêmica, relacionada a uma mutação Xq22 no cromossomo X, que resulta em deficiência da enzima alfa-galactosidase, diminuindo a capacidade de degradação da globotriaosilceramida. **Objetivos:** avaliar a prevalência de mutações na doença de Fabry, bem como seus sinais e sintomas, em familiares de pacientes com doença renal crônica (DRC) diagnosticados com DF durante um estudo realizado anteriormente, denominado “Análise clínica e epidemiológica da doença de Fabry em centros de diálise no Brasil”.

**Métodos:**

foi realizado um estudo transversal e os dados foram coletados através da entrevista com familiares de pacientes inscritos no Projeto Rim Fabry Brasil e exames de sangue para dosagem de Gb3 e testes genéticos.

**Resultados:**

Dos 1,214 familiares entrevistados, 115 (9,47%) foram diagnosticados com DF, com predomínio de mulheres (66,10%). As comorbidades mais prevalentes foram condições reumatológicas e hipertensão arterial sistêmica (1,7% cada), seguidas por doenças cardíacas, neurológicas, cerebrovasculares e depressão em 0,9% dos indivíduos. Intolerância ao exercício físico e cansaço foram os sintomas mais observados (1,7%), seguidos de febre periódica, intolerância ao calor ou ao frio, dor difusa, sensação de queimação ou dormência nas mãos e nos pés, sudorese reduzida ou ausente, além de dor abdominal após refeições em 0,9%.

**Conclusão:**

Encontramos uma prevalência da doença de Fabry em 9,47% dos familiares de pacientes com DRC com essa condição, notadamente com uma predominância de 66,1% de mulheres, o que contrasta com relatos anteriores. A triagem de familiares de pacientes com DF é importante, pois pode levar ao diagnóstico e tratamento precoces, permitindo melhor qualidade de vida e melhores resultados clínicos para esses indivíduos.

## INTRODUCTION

Fabry disease (FD) (OMIM 301500) is caused by a deficiency of the enzyme alpha galactosidase (-Gal-A, EC 3.2.1.22), which results in impaired clearance of globotriaosylceramide (Gb3)^1^. FD is a progressive, multi-system, and hereditary condition, linked to a mutation in the Xq22 region of X chromosomes^2^^,^ 3 ^,^ 4. It is considered a rare or “orphan” disease, due to its very low prevalence^2^^,^ 4. Gb3 accumulates within lysosomes throughout the whole body, notably the brain, heart, kidneys, nervous system, and skin, leading to several signs and symptoms and substantial morbidity and mortality^3^^-^ 6.

The first symptoms of FD, such as acroparesthesia, abdominal pain, especially following meals, and diarrhea, usually occur in the first decade of life, but are very nonspecific, thus leading to late medical referral and diagnosis^5^^-^ 7. Angiokeratomas are the most characteristic clinical signs (Germain 2010; Hopkin et al 2008). FD can cause left ventricular hypertrophy (LVH), stroke and transient ischemic attacks, hearing loss, cornea verticilata, development of proteinuria, and progressive kidney disease^4^^-^ 6 ^,^ 8.

The incidence of FD varies from 1:40,000 to 1:117,000, and although prevalence can vary according to different regions, no precise ethnical predisposition is known to date^3^^,^ 4 ^,^ 6. Studies reporting the prevalence of FD in patients with chronic kidney disease are more frequent^9^^-^ 19. Few studies have evaluated the relatives of patients with FD^14^^-^ 16 ^,^ 18 ^,^ 20 ^,^ 21. The only Brazilian study to evaluate relatives of FD patients found a prevalence of 0.12% of FD in men undergoing hemodialysis (HD)^20^. 

In men, diagnosis is made by demonstration of low enzymatic activity of α-Gal-A. In women, genetic sequencing is necessary due to the presence of two X chromosomes, hence measured α-Gal-A activity is almost always normal^6^^,^ 8 ^,^ 22. A number of studies reveal different prevalence rates of FD among relatives of patients diagnosed with FD, pointing to the importance of early diagnosis, in order to allow better handling of the disease^7^^,^ 12 ^,^ 14 ^-^ 16 ^,^ 18 ^,^ 20 ^,^ 21.

The goal of the present study was to evaluate the prevalence of FD mutations and to describe the presence of signs and symptoms of this disease among relatives of FD mutations patients from the previously undertaken study entitled “Clinical and epidemiological analysis of Fabry disease in dialysis centers in Brazil - the Brazil Fabry Kidney Project”^12^.

## MATERIAL AND METHODS

This was a cross-sectional study, approved by the local ethics committee under the number 18029513.0.0000.5244. 

**Figure 1:**
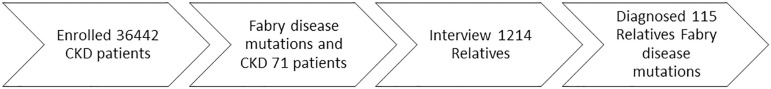
Study design.

Initially, of a total 36,442 patients with chronic kidney disease (CKD) enrolled in the Brazil Fabry Kidney project, 71 were diagnosed with FD mutations. Through medical anamnesis, 1214 relatives of those FD patients were identified as possible FD carriers, and offered informed consent agreements to continue the study through blood sampling for enzymatic dosages and genetic testing (Figure 1). No financial gain was offered to participants, and the study followed the regulations stated in the declaration of Helsinki.

Blood sampling was made through paper filters, to allow dosage of α-Gal-A by tandem mass spectrometry, a screening method with sensitivity and specificity of around 96% (Centogene AG; Schillingallee 68; 18057 Rostock, Germany). When enzymatic activity was low (<2.6 µmol/L/h) or absent, DNA sampling was performed to confirm the diagnosis of FD mutation. For the genetic testing, PCR analysis of the GLA gene, as well as sequencing of the whole coding region and of the highly conserved exon-intron junction regions were performed. The GLA gene was analyzed by next generation sequencing based on amplicon. Amplicons cover the entire coding region and highly conserved splicing junctions. A minimum coverage of >20x was obtained for each amplicon. The reference sequence is: NM_000169.2. The classification of the variants was based on the recommendations of the American College of Medical Genetics. (Centogene AG; Schillingallee 68; 18057 Rostock; Germany).

The FD mutations prevalence was calculated and a descriptive data analysis was performed.

## RESULTS

**Figure 2:**
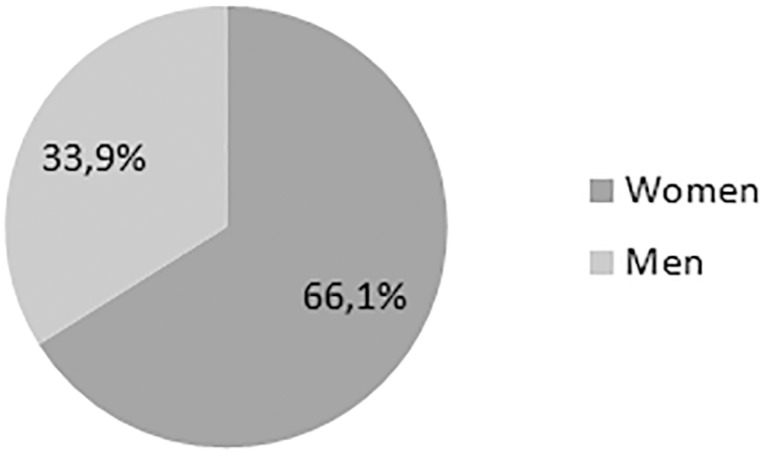
Gender proportion of patient’s relatives diagnosed with Fabry disease mutations.

**Figure 3:**
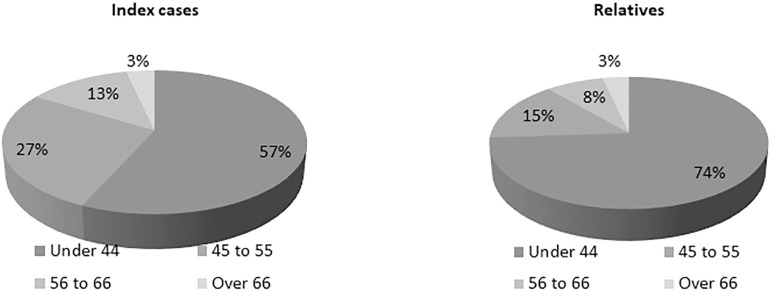
Sample proportions of Fabry disease mutations diagnosis by age.

The prevalence of FD mutations in the studied population was 9.47% (115 of 1214), of which 66.1% were women (Figure 2). Diagnosis of FD mutations was made in people younger than 44 years in 74% of participants (Figure 3). There was no difference between age and gender regarding the prevalence of mutations (Table 1).

**Table 1:** Sample proportions of Fabry disease mutations (DF) diagnosis by gender and age.

Gender Age	Relatives N 1214 (%)	Relatives DF N 115 (%)	Index Cases DF N 71 (%)
Female	657 (54.1%)	76 (66.1%)	45 (63.4%)
Male	557 (45.9%)	39 (33.9%)	26 (36.6%)
Less than 44 years old	385 (31.7%)	85 (73.9%)	40 (56.3%)
45 to 55 years old	353 (29.1%)	17 (14.8%)	19 (26.8%)
56 to 66 years old	218 (18%)	9 (7.8%)	9 (12.7%)
More than 66 years old	258 (21.3%)	4 (3.5)	3 (4.2%)
Percentage average	50.51 %	33.15 %	41.42 %

N= number.

Most of the relatives were asymptomatic and the most common comorbidities were rheumatologic conditions and systemic hypertension (1.7% each). Neurological, heart and cerebrovascular conditions, and depression were found in 0.9% of subjects (Table 2). Regarding signs and symptoms, intolerance to physical exercise and tiredness (1.7%), followed by periodic fever, intolerance to heat or cold, diffuse pain, burn sensation or numbness in hands and feet, reduced or absent sweating, as well as abdominal pain after meals was observed in 0.9% (Table 3).

**Table 2:** Comorbidities observed in index cases and relatives with Fabry disease mutations.

Comorbidities	Total N 186 (%)	Index cases N 71 (%)	Relatives N 115 (%)
Rheumatologic conditions	28.5	71.8	1.7
Systemic hypertension	14.5	35.2	1.7
Heart disease	23.7	60.6	0.9
Depression	11.3	28.2	0.9
Neurological disease	10.2	25.4	0.9
Cerebrovascular disease	10.2	25.4	0.9
Dermatological conditions	4.8	12.7	0
Diabetes	3.2	8.5	0
Ophthalmological conditions	2.7	7	0
Obesity	1.6	4.2	0
Policystic kidneys	1.1	2.8	0
Berger disease	0	0	0

N= number.

**Table 3:** Sign and symptoms observed in index cases and relatives with Fabry disease mutations.

Signs and symptoms	Total N 186 (%)	Index cases N 71 (%)	Relatives N 115 (%)
Intolerance to exercise or tiredness	8.1	18.3	1.7
Periodic fever	7	16.9	0.9
Intolerance to heat or cold	11.3	28.2	0.9
Burning sensation in hands and feet	9.7	23.9	0.9
Spreading pain throughout the body	10.8	26.8	0.9
Numbness in hands and feet	9.1	22.5	0.9
Reduced or absent sweating	16.7	42.3	0.9
Abdominal pain after meals	9.7	23.9	0.9
Hearing impairment	9.1	23.9	0
Diarrhea after meals	0	0	0
Cornea verticilata	2.7	7	0
Angiokeratomas	4.8	12.7	0

N= number.

## DISCUSSION

The goal of this study was to evaluate the prevalence of FD mutations and observe the presence of signs and symptoms related to FD among relatives of CKD patients with FD mutations, as a secondary analysis related to a previous study^12^. We found a high prevalence of FD mutations among those relatives (9.47%), which confirmed the hypothesis that this particular population is at high risk for FD mutations^14^^-^ 16 ^,^ 21. Our data contrasted with a number of previous reports, which had showed the prevalence of FD in relatives of previously diagnosed FD patients to be lower than 1.8%^15^^-^ 17. Due to the rarity of this condition, other studies only described numbers of cases, without defining the prevalence of FD in relatives of FD patients, as is the case of one Latin American (24 cases)^21^, three European (23, 8 and 11 cases)^14^^-^ 16, and one Brazilian study (23 cases)^20^.

The most observed comorbidities in our study were rheumatologic conditions and systemic hypertension, in 1.7% of individuals, followed by neurological, cerebrovascular and cardiac conditions, and depression, in 0.9%. Another previous Latin American study described kidney disease, stroke, left ventricle hypertrophy, and deafness as the most common observed conditions^21^.

Our study shows similar percentages to the European and Asian studies for systemic hypertension, history of heart disease and stroke, differing in relation to diabetes, renal involvement, cardiac arrhythmia, and cardiomyopathic hypertrophy^14^^-^ 16 ^,^ 18. Compared to the Brazilian study by Silva (2016), the percentages of left ventricular hypertrophy and systemic hypertension were similar, differing only in Fabry’s nephropathy^20^.

We observed a low prevalence of signs and symptoms, and the most common were intolerance to physical exercise and tiredness, in 1.7%, followed by periodic fever, intolerance to heat or cold, diffuse pain, burn sensation or numbness in hands and feet, reduced or absent sweating, as well as abdominal pain after meals, in 0.9% of individuals, which highlights the importance of early diagnosis, allowing recognition of FD cases prior to marked systemic derangement due to more severe accumulation of Gb3^7^^,^ 21.

Previously, one Latin American study found angiokeratomas, hypohidrosis or anhidrosis, impaired temperature regulation with intolerance to cold and heat, acroparesthesias, cornea verticilata, diarrhea or constipation, abdominal pain, deafness, tinnitus and fatigue in patients and relatives, but there was no analysis evaluating the groups separately^21^. Okur et al, in 2013, described two cases of FD in patients on dialysis: in case 1, the patient’s granddaughter presented cornea verticilata, and, in case 2, one relative presented proteinuria, while another presented acroparestesia and cornea verticilata^13^. A Turkish study identified in a family screening eight relatives of a FD patient to have FD, and described multiple symptoms in 75% of these^15^. Another Turkish study described hearing loss, intolerance to heat and cold, and sweating disorders to be the most commonly observed disorders in patients, however, in spite of having found 3 cases of FD between relatives, there was no description of the clinical manifestations^17^. Finally, in Asia, the most typically described features in FD patients were sweating impairment (73.5%), corneal clouding (73.0%), and acroparesthesia (72.2%); although ten family members presented mutations to FD, there was no description of their signs and symptoms^18^.

In the aforementioned Brazilian study, most cases presented classical features of FD: cornea verticilata, hypohidrosis, acroparesthesia, angiokeratoma, lymphedema, arthralgia, generalized pains and fatigue, which was similar to our findings, whereas their relatives were mostly oligosymptomatic, hence would clearly benefit from early diagnosis and treatment^20^. In our study, the majority of relatives were oligosymptomatic and 73.9% of relatives were diagnosed with FD mutations earlier than 44 years of age, similarly to other studies in which genetic testing allowed early recognition of the disease^15^^,^ 20 ^,^ 21.

Among relatives of FD mutation patients, we found a proportion of 66.1% of women to be diagnosed with FD mutations, which is very different from what was previously shown in the literature, since women had rarely been screened for FD^15^^,^ 17 ^,^ 21. Most studies report a higher prevalence of FD in men, probably due to higher screening in men; in our opinion, women should definitely also be screened^9^^-^ 12 ^,^ 19. In fact, as in men, women can develop classical forms of FD, and as demonstrated in a previous Latin American study, heterozygous women can show various degrees of disease severity^21^.

The main limitation of the study was that we did not have the family tree of the patients, which is inherent to our study design. Another limitation was that the levels of alpha galactosidase were not available in this database. These limitations do not invalidate our relevant study.

Screening of FD in men consists of dosage of α-Gal-A, which is much cheaper than genetic testing necessary for diagnosis of FD in women^8^^,^ 22. Screening for FD is of great importance, since life expectancy can be diminished in roughly 20 years in FD patients, with marked reduction in survival in men aged over 35 years, whereas clinical manifestations tend to occur later in women, with an estimated 15 years loss in life expectancy^6^^,^ 8.

## CONCLUSION

We found a prevalence of FD mutations in 9.47% of relatives of CKD patients diagnosed with FD mutations, 66.1% of which were women. Our results suggest that relative screening of FD mutations, in both men and women, is necessary, not only due to the high prevalence of FD mutations found in this study, but also because FD can be oligosymptomatic for many years, meaning that early diagnosis can favor treatment success and prevention of severe complications of multi-system Gb3 deposition. Women, even though relatively protected by the presence of two X chromosomes, are susceptible to FD, and screening of this disease has often been neglected in women, possibly due to the higher costs of genetic sequencing, when compared to enzymatic dosage for diagnosing FD in men.
